# Seroprevalence of *Brucella suis* in eastern Latvian wild boars (*Sus scrofa*)

**DOI:** 10.1186/s13028-018-0373-9

**Published:** 2018-03-24

**Authors:** Lelde Grantina-Ievina, Jelena Avsejenko, Svetlana Cvetkova, Dita Krastina, Madara Streikisa, Zanete Steingolde, Indra Vevere, Ieva Rodze

**Affiliations:** Animal Disease Diagnostic Laboratory, Institute of Food Safety, Animal Health and Environment “BIOR”, 3 Lejupes Street, Riga, 1076 Latvia

**Keywords:** *Brucella suis*, Brucellosis, Latvia, *Sus scrofa*, Wild boar

## Abstract

Brucellosis due to *Brucella suis* biovar 2 is one of the most important endemic diseases in wild boar (*Sus scrofa*) populations in Europe. The aim of the present study was to determine the seroprevalence of brucellosis in wild boars in the eastern part of Latvia. Wild boars killed by hunters in the period from January to April 2015 (n = 877) and from March to April in 2016 (n = 167) were examined for antibodies against *B. suis* by the Rose Bengal test (RBT), a complement fixation test (CFT), and by enzyme-linked immunosorbent assays. In 2015, 199 samples (22.7%) were positive by RBT and/or CFT while 36 samples (21.6%) were seropositive in 2016. Of the *Brucella* seropositive samples from 2015 and 2016 (n = 235), 162 (68.9%) were also seropositive to *Yersinia enterocolitica.* Considering cross-reactivity of serological tests, the seroprevalence of *B. suis* biovar 2 exposure in wild boars in the eastern part of Latvia was calculated to 14.0% in 2015 and 9.6% in 2016. From selected seropositive samples (42 in 2015 and 36 in 2016) total DNA was extracted and analyzed with an IS*711*-based nested polymerase chain reaction (PCR) assay. Species and biovar identification was conducted for bacteria isolated in monoculture from PCR positive samples by species specific primers and Bruce-ladder multiplex PCR. *Brucella suis* biovar 2 was isolated from 12/20 samples in 2015 and 9/9 samples in 2016. The average seroprevalence was relatively low compared to that found in certain other European countries. Males and females had an equal level of seropositivity, but a positive age-trend was observed for both males and females.

## Findings

Porcine brucellosis can be caused by three biovars (1–3) of *Brucella suis*. Biovar 2 is an important pathogen in wild boars (*Sus scrofa*) with a broad geographical distribution ranging from Scandinavia to the Balkan region [1]. Systematic brucellosis monitoring in wildlife is not demanded by regulatory acts but several studies have reported the presence of this infection in European countries [2–9]. Scientific data on the prevalence of *B. suis* biovar 2 in the Baltic countries, Russia and Belorussia have not been published. A few cases of domestic pig brucellosis have been recorded in Estonia (2006) and Latvia (2007 and 2008) [10]. The latest outbreak in Latvia was in 2010 in the western part of the country (unpublished observations).

Transmission of *Brucella* bacteria occurs during copulation and by consumption of infected birth and abortion products and uterine discharges. Infection is not necessarily associated with the presence of gross lesions [11]. Wild boars as well as the European hare (*Lepus capensis*) are considered as reservoirs for transmissions of *B. suis* biovar 2 to domestic livestock [1], mainly due to consumption of offal from hunted or dead infected hares by wild boars [10].

According to estimates made by the Latvian State Forest Service, the population of wild boars in Latvia increased during the last decades from around 15,000 in 1997 to 74,000 in 2013, but decreased to 49,000 in 2015 due to promoted hunting. The estimated population of European hares in Latvia is 34,700, indicating the potential for transmission of the infection from this host [12].

The aim of the present study was to determine the seroprevalence of brucellosis in wild boars in the eastern part of Latvia and its correlation to gender and age.

Blood and tissue samples (spleen, kidney, tonsil and lymph nodes) were collected from wild boars killed by hunters from January to April 2015 (n = 877) and from March to April 2016 (n = 167) conducted within the national surveillance programs aimed on African and classical swine fever viruses. Hunters determined gender of the animals and age based on tooth eruption pattern (< 12, 12–24, > 24 months). All tested animals were evaluated as clinically healthy by hunters and veterinarians, i.e. no obvious clinical or pathological signs of brucellosis were observed.

Samples were transported to the laboratory refrigerated at 4 °C. Blood samples were transferred to 5 ml tubes, centrifuged and kept at 4 °C until analysis but not longer than 5 days. The tissue samples were kept at − 20 °C until analysis.

Sera were tested by the Rose Bengal test (RBT) (Rose Bengal assay, IDEXX, Westbrook, USA) and a complement fixation test (CFT) according to the OIE Manual of Diagnostic Tests and Vaccines for Terrestrial Animals [1] and Standard Operating Procedures of European Union Reference Laboratory for Brucellosis [13, 14]. For the CFT, the following compounds were used: *Brucella* antigens (IDEXX or IDvet, Grabels, France), sheep blood in Alsvers (TCS Biosciences Ltd, Botolph Claydon, UK), rabbit haemolytic serum (TCS Biosciences Ltd), calcium–magnesium veronal buffer (IDvet) and guinea pig complement (IDvet). Samples were recorded as seropositive if either the RBT and/or CFT was positive, and these were further tested with indirect enzyme-linked immunosorbent assays (ELISAs) (Ingezim Brucella porcina, Ingenasa, Madrid, Spain) and screened for presence of *Yersinia enterocolitica* antibodies by an indirect ELISA (Pigtype Yopscreen, Labor Diagnostik Leipzig, Leipzig, Germany).

To identify individuals suitable for *Brucella* isolation, i.e. animals with an expected high bacterial load, total DNA was extracted from 78 pooled tissue specimens of seropositive animals (strong positive RBT (≥ 1+) and/or CFT (≥ 23.33 IU/ml); n = 42 in 2015 and n = 36 in 2016). Extracted DNA was subjected to IS*711*-based nested polymerase chain reaction (PCR) [15]. In the case of positive PCR, tissues from 20 animals in 2015 and nine animals in 2016 were subjected to bacteriological culturing. Culturing was done on spleen, kidney, tonsil and lymph nodes separately, according to [1, 16]. Biovar determination was done by further cultivation on selective agar with/without CO_2_, the H_2_S test, growth in the presence of dyes (thionin and basic fuchsin), slide agglutination tests with monospecific A, M, R antisera and lysis by phages according to [1, 17]. These tests were followed by species and biovar confirmation with species specific PCR [18] and Bruce-ladder multiplex PCR [19].

Statistical analyses were done using R program [20] and Chi square test [21]. True prevalence was calculated using EpiTools epidemiological calculators [22].

In 2015, the sampling area covered 40 municipalities with a total area of 30,177 km^2^. A total of 199 animals [22.7%; 95% confidence interval (CI) 20.04–25.58] were seropositive for *Brucella*. In 2016, the sampling area covered 18 regional municipalities with a total area of 18,461 km^2^. Of the 167 tested animals, 36 (21.6%; CI 16.0–28.4) were serologically positive for *Brucella*. Data of both study years are combined in Table 1. In 2015, 130 (65.3%) of the seropositive animals were also seropositive to *Y. enterocolitica*, while this was the case for 32 (88.9%) animals in 2016 (Table 2). Due to cross-reactivity between *B. suis* and *Y. enterocolitica* in serological tests, serology data were combined with PCR results revealing a prevalence of *B. suis* biovar 2 infection of 14.0 and 9.6% in 2015 and 2016, respectively. This level of exposure to *B. suis* is relatively low compared to that in certain other European countries. Serological surveys have reported the proportion of seropositive animals estimated by microagglutination test and CFT to be as high as 15.0% in the Czech Republic (1995–1996) [3], while estimations using RBT and CFT were 19.7% in Italy (2001–2007) [6], and 22.6–29.4% in Croatia (1996–2000), and estimations based on ELISA were 22.0% in north-eastern Germany (1995–1996) [5], up to 39.6% in some cantons of Switzerland (2001–2003) [8], and on average 24.4% in Poland (2012) [9]. Among these investigations the cross-reactivity problem of serological tests with *Y. enterocolitica* was assessed only in Germany [5].

**Table 1:** Serological prevalence for *Brucella suis* in the wild boar population in the eastern part of Latvia in 2015–2016

Gender	Age category								Total	
	< 12 months		12–24 months		> 24 months		Unknown			
	Number of animals/positive	Prevalence (%)	Number of animals/positive	Prevalence (%)	Number of animals/positive	Prevalence (%)	Number of animals/positive	Prevalence (%)	Number of animals/positive	Prevalence (%)
Male	154/21	13.64	205/47	22.93	214/62	30.53	0/0	0.00	573/130	22.69
Female	127/18	14.17	184/40	21.74	73/19	27.87	1/0	0.00	385/77	20.00
Unknown	5/1	20.00	3/3	100.00	3/3	100.00	75/21	28.00	86/28	32.56
Total	286/40	13.99	392/90	22.96	290/84	30.71	76/21	27.63	1044/235	22.51

**Table 2:** Comparison of nested polymerase chain reaction results with the Rose Bengal test, complement fixation test, and indirect enzyme-linked immunosorbent assays of swine brucellosis and *Y. enterocolitica* in 2015 (n = 42) and 2016 (n = 36)

PCR result	RBT 2015/2016		CFT 2015/2016		ELISA 2015/2016		ELISA *Y. enterocolitica* 2015/2016	
	Pos	Neg	Pos	Neg	Pos	Neg	Pos	Neg
Positive	24/18	NT	19/16	5/2	23/18	1/0	10/16	14/2
Negative	18/18	NT	18/9	NT/7	18/14	NT/4	16/15	2/2

RBT, Rose Bengal test; CFT, complement fixation test; ELISA, Ingezim Brucella porcina; ELISA *Y. enterocolitica*, Pigtype Yopscreen; Pos, positive; Neg, negative; NT, not tested

**Fig. 1:**
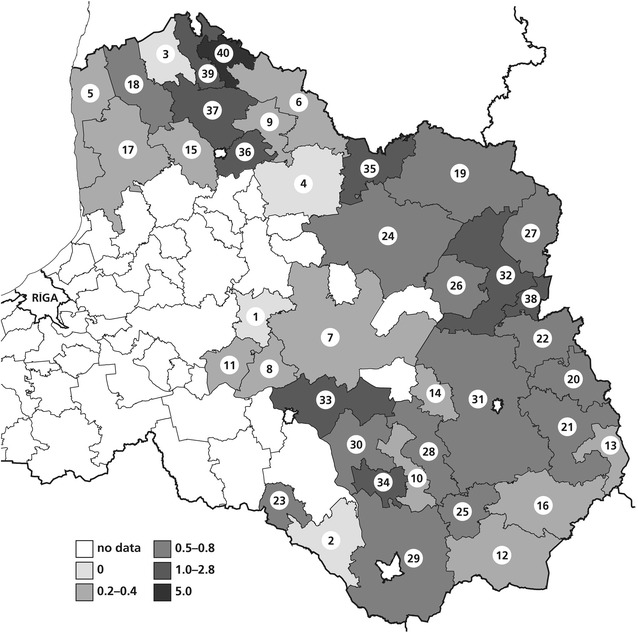
Average density of infected wild boars estimated as serologically positive animals per 100 km^2^ (2015). Regions: 1—Erglu, 2—Ilukstes, 3—Mazsalacas, 4—Smiltenes, 5—Salacgrivas, 6—Valkas, 7—Madonas, 8—Plavinu, 9—Strencu, 10—Preilu, 11—Kokneses, 12—Kraslavas, 13—Zilupes, 14—Vilanu, 15—Kocenu, 16—Dagdas, 17—Limbazu, 18—Alojas, 19—Aluksnes, 20—Ciblas, 21—Ludzas, 22—Karsavas, 23—Aknistes, 24—Gulbenes, 25—Aglonas, 26—Rugaju, 27—Vilakas, 28—Riebinu, 29—Daugavpils, 30—Livanu, 31—Rezeknes, 32—Balvu, 33—Krustpils, 34—Varkavas, 35—Apes, 36—Beverinas, 37—Burtnieku, 38—Baltinavas, 39—Rujienas, 40—Nauksenu

The density of seropositive wild boars in 2015 ranged from 0 to 5 animals per 100 km^2^ (Fig. 1). The number of tested animals ranged from 0.2 to 12 animals per 100 km^2^. The regions with the highest numbers of serologically positive animals (2–5 per 100 km^2^) where those with relatively high numbers of hunted and tested animals. The seroprevalence in these regions ranged from 25.0% (in Baltinavas and Rujienas regions) to 42.4% (Nauksenu region). The density of seropositive wild boars in 2016 ranged from 0 to 0.5 animals per 100 km^2^. The number of tested animals ranged from 0.1 to 3.8 animals per 100 km^2^ (data not shown).

In 2015, 24 pooled tissue samples from 42 seropositive animals were positive by the IS*711*-based nested PCR assay (Table 2) and *B. suis* biovar 2 was isolated from 12 of 20 samples, of which 15 originated from PCR positive animals. In most of the cases, isolation from the spleen samples was successful. In 2016, 18 of 36 tissue samples from seropositive animals were positive for IS*711* by PCR. *B. suis* biovar 2 was cultured from 9 of 9 selected PCR positive samples.

The prevalence of seropositive boars did not differ between genders. The prevalence of seropositive boars was positively correlated with increased age irrespectively of gender for 2015 samples (χ^2^ = 14.6, P = 0.0007 for males and χ^2^ = 6.26, P = 0.04 for females). Positively correlated seroprevalence among age groups have been observed also in other countries, for example, in Italy [6], and a sex/age interaction was found in Spain [23]. In 2016 differences between age categories were statistically not significant probably due to the low number of tested animals in this year.

Statistically significant differences were obtained also by sampling month in 2015 (χ^2^ = 17.6, P = 0.0005) with the highest prevalence in April (36.8%). Significant differences among sampling seasons have been recorded also in other investigations, for example, in Spain in relation to hunting activities [23].

The seroprevalence in some regions of eastern Latvia was 25.0–42.4%. These areas corresponded to regions with the highest percentage of forest area (57%) compared to the average forest area of 50% in Latvia in general [12]. A high degree of forest area is probably positively correlated with a high density of wild boars. It has been estimated that 89% of the territory of Latvia contains habitat suitable for wild boars [24]. The average seroprevalence for *B. suis* in Latvian wild boars seems to be relatively low in comparison to certain other European countries but still the wild boar population has to be considered as an important reservoir for the *B. suis* biovar 2 transmission to domestic pigs.

## Abbreviations

CI: confidence interval
CFT: complement fixation test
ELISA: enzyme-linked immunosorbent assay
LPS: lipopolysaccharide
NT: not tested
PCR: polymerase chain reaction
RBT: Rose Bengal test
